# The Governing Role of Si/Al Ratio in the Structural Evolution and Mechanical Properties of N-A-S-H Gel

**DOI:** 10.3390/ma19020246

**Published:** 2026-01-07

**Authors:** Min Hu, Jiayun Chen, Bo Xia, Jiejin Chen

**Affiliations:** 1 School of Civil and Environmental Engineering, Changsha University of Science and Technology, Changsha 410114, China; chenjiayun@csust.edu.cn; 2 Hunan Provincial Engineering Research Center for Bridge Engineering Safety Control Technology and Equipment, Changsha 410114, China; 3 School of Hydraulic and Ocean Engineering, Changsha University of Science and Technology, Changsha 410114, China; boxia@csust.edu.cn

**Keywords:** alkali-activated cementitious materials, molecular dynamics, low-calcium fly ash, N-A-S-H gel, reaxff

## Abstract

Alkali-activated cementitious materials are environmentally friendly alternatives to traditional cement. The structure of their core product, sodium aluminosilicate hydrate (N-A-S-H) gel, is regulated by the silicon-to-aluminum (Si/Al) ratio; however, the atomic-scale mechanism underlying this influence remains unclear. Integrating reactive force field molecular dynamics simulations and experiments, this study systematically reveals the regulation mechanism of the Si/Al ratio (1.0–2.0) on the microstructure and macroscopic properties of N-A-S-H gels. Starting from well-defined PS and PSS oligomers, the simulation results demonstrate that the Si/Al ratio governs the polymerization pathway, aluminum coordination environment (especially the content of pentacoordinate aluminum), and evolution of nanoporosity. When the Si/Al ratio is approximately 1.8, the system exhibits the highest silicate polymerization degree, lowest nanoporosity, and densest three-dimensional (3D) network structure; deviation from this ratio leads to structural degradation due to charge imbalance or excessive polymerization. These computational findings are validated by experiments on fly ash-based geopolymers: the material achieves the highest compressive strength at a Si/Al ratio of 1.8. The consistency between simulations and experiments collectively reveals a cross-scale action mechanism: the Si/Al ratio determines the macroscopic mechanical properties by regulating the nanoscale packing density and defect distribution of the gel. This study provides critical atomic-scale insights for the rational design of high-performance geopolymers.

## 1. Introduction

Geopolymers, as inorganic polymeric materials formed by dissolution-condensation reactions of alkali-activated aluminosilicate precursors (e.g., low-calcium fly ash, metakaolin), are widely regarded as one of the most promising sustainable cementitious materials to replace traditional Portland cement due to their excellent mechanical properties, outstanding durability, and significantly reduced carbon emissions [1,2]. Their core performance stems from the sodium aluminosilicate hydrate (N-A-S-H) gel as the binding phase, which is a three-dimensional amorphous network structure composed of [SiO_4_]^4−^ and [AlO_4_]^5−^ tetrahedra connected by shared oxygen atoms [3,4]. However, N-A-S-H gel is inherently amorphous with long-range disorder, and the structure-activity relationship between its microstructure (e.g., local order, aluminum coordination environment, nanoscale pores) and macroscopic properties remains a key scientific bottleneck restricting the rational design and performance optimization of geopolymer materials [5].

Among numerous regulatory factors, the molar silica-alumina ratio (Si/Al) of the system is a core parameter affecting the formation and final performance of N-A-S-H gel, directly determining the setting time, mechanical strength, and durability of the material [6]. Numerous experiments have shown that macroscopic properties such as compressive strength first increase and then decrease with the change in Si/Al ratio, with an optimal value in the range of 1.5–2.0 [7,8,9,10]. Although characterization techniques such as X-ray diffraction (XRD), Fourier transform infrared spectroscopy (FTIR), scanning electron microscopy (SEM), and mercury intrusion porosimetry (MIP) can provide information on phase composition, chemical bonds, microstructure, and pore structure [11,12,13], it is difficult to dynamically capture the details of structural evolution at the atomic/nanoscale, such as the distribution of aluminum coordination states, condensation reaction pathways, and the formation process of nanoscale pores. Therefore, the microscopic mechanism by which Si/Al affects macroscopic properties remains unclear.

With the development of reactive force fields (ReaxFF), molecular dynamics (MD) simulation has become an important means to explore the gel formation mechanism at the atomic scale. It can effectively simulate chemical reaction processes such as hydrolysis and condensation [14,15,16,17,18], and has been used to investigate the influence of Si/Al ratio on the microstructure [11], thermal conductivity [19], and mechanical properties [20,21] of gels. However, most existing simulations start from “averaged” amorphous macromolecules or oligomers, failing to dynamically simulate their assembly process in competitive condensation based on well-defined structural units (e.g., PS, PSS). Thus, it is difficult to understand how Si/Al regulates network topology from the source of the reaction, and it is impossible to accurately explain the correlation mechanism between microstructure evolution and macroscopic properties.

The structural unit model proposed by Davidovits (e.g., PS, PSS) provides a classic framework for describing the short-range order of gels [22]. In recent years, advanced characterization techniques such as solid-state nuclear magnetic resonance (NMR) have provided experimental evidence for verifying the evolution of different structural units with Si/Al ratio [23,24], and SEM-EDS, XRD, and FTIR characterizations have also confirmed the influence of Si/Al ratio on the formation of structural units [25]. These advances indicate that starting from basic units with clear chemical definitions such as PS and PSS, adopting a bottom-up simulation approach to dynamically track their condensation and assembly processes will be more conducive to revealing the regulatory mechanism of Si/Al ratio on the structure of N-A-S-H gel.

To this end, this study designed a closed-loop research combining molecular dynamics simulation and multi-scale experimental verification. In terms of simulation, the validated ReaxFF reactive force field was adopted, with stoichiometrically precise PS (Si/Al = 1) and PSS (Si/Al = 2) as initial species to dynamically simulate their competitive condensation and gelation processes, so as to reveal the influence mechanism of Si/Al ratio on network atomic packing, connection topology, and nanoscale pores. In terms of experiments, alkali-activated low-calcium fly ash specimens with different Si/Al ratios were prepared by adjusting the ratio of glass powder to sodium silicate, and characterized by SEM, XRD, and mechanical tests. Based on the analysis of structural evolution at the atomic scale, this study aims to clarify the microscopic mechanism by which Si/Al ratio regulates the formation and performance optimization of N-A-S-H gel, and provide theoretical and data support for the rational design and performance improvement of high-performance geopolymers.

## 2. Materials and Methods

### 2.1. Molecular Dynamics Simulation

#### 2.1.1. Simulation Strategy and Model Construction

**Figure 1:**
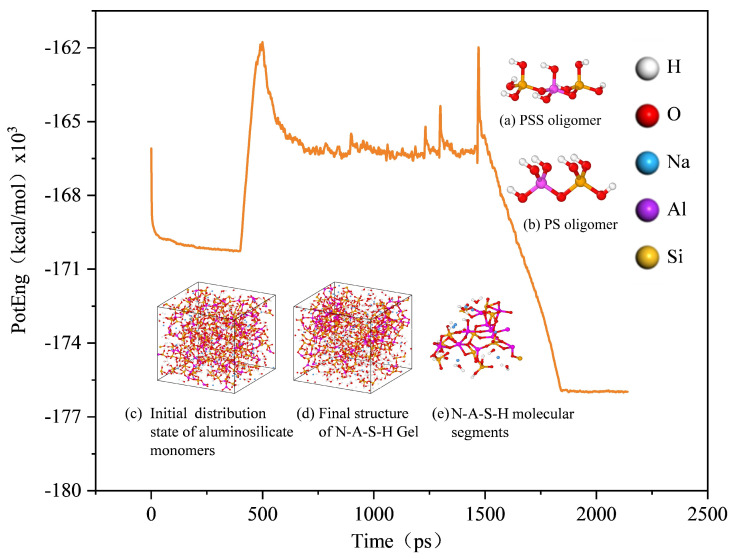
Variation in system potential energy during simulation, Network Structure of N-A-S-H Gel and PS, PSS.

Based on the structural unit model proposed by Davidovits [22], this study adopted a bottom-up strategy to construct gel models. Polyaluminosilicate (PS, SiAlO_7_H_6_, Si/Al = 1) and polyaluminosiloxane (PSS, Si_2_AlO_10_H_8_, Si/Al = 2) oligomers were employed as the basic building units, and examples of PS and PSS oligomers are shown in Figure 1a and Figure 1b, respectively. By precisely controlling the quantity ratio of the two oligomers, four initial systems with target Si/Al ratios (1.0, 1.5, 1.8, 2.0) were established in cubic periodic boundary boxes (side length: 32.5 Å) using Materials Studio 2020 (20.1.0.2728). A corresponding number of Na^+^ ions (from NaOH) was added to each system to balance the charge of [AlO_4_]^5−^ tetrahedra generated from oligomer hydrolysis, and sufficient water molecules were introduced (with a mass ratio of water to cementitious materials of 0.4:1) to simulate a high-alkalinity solution environment, ensuring the full dissolution and migration of reactants. The four constructed model systems (with Si/Al ratios of 1.0, 1.5, 1.8 and 2.0, respectively) had initial densities ranging from 1.44 to 1.97 g/cm^3^, which increased systematically with the increase in the Si/Al ratio and is consistent with the actual concentration range of alkali-activated pastes. The detailed initial atomic compositions, box sizes and key parameters are listed in Table 1.

#### 2.1.2. Force Field Selection and Simulation Details

The Reactive Force Field (ReaxFF) developed by van Duin et al. [26,27] was adopted for simulations. Based on the bond order concept, this force field can dynamically describe the formation and cleavage of chemical bonds, making it suitable for simulating condensation reactions in alkali-activated environments. Its potential energy function incorporates bond order-dependent bond energy, bond angle, dihedral angle, van der Waals, and Coulomb interaction terms. The ReaxFF parameters for Na, Si, Al, O, and H were derived from previously published results [27,28,29,30], whose reliability has been fully validated.

Molecular dynamics simulations were performed using the LAMMPS software (4 February 2025) with a time step set to 0.25 fs. The simulation procedure (see Table 2) was designed to drive the system from dispersed oligomers to condensed gel networks, following four sequential steps: first, energy minimization was conducted to eliminate unreasonable configurations; subsequently, NVT and NPT ensemble equilibration were carried out at 300 K to stabilize the initial structure and adjust density; then, the system was rapidly heated to 2000 K under NVT ensemble to provide kinetic energy for overcoming reaction barriers, followed by sufficient relaxation at this temperature to facilitate polymerization; finally, rapid quenching to 300 K and subsequent equilibration were performed to allow gel structure reorganization and stabilization.

This procedure balances simulation efficiency and physical realism: high-temperature relaxation accelerates the reaction, while subsequent quenching and low-temperature equilibration facilitate the formation of thermodynamically more stable structures. When the system reaches a local energy minimum state, its structural characteristics remain stable [31]. Though the limited model size affects long-range structural statistics, this approach is sufficient to capture the key dynamic processes of N-A-S-H gel formation (e.g., ion association, chemical bond formation, and local structural relaxation). Through the above procedure, well-equilibrated N-A-S-H gel structures were obtained, as illustrated in Figure 1c–e.

#### 2.1.3. Structural Analysis and Characterization Methods

Quantitative analysis and visualization of the final equilibrated trajectories of the systems were performed using OVITO software 3.12.1, including specifically: (1) evaluating the polymerization degree by calculating the oxygen coordination numbers of individual Si and Al atoms to statistically analyze the distributions of Si^0^–Si^4^ and Al^0^–Al^6^ species; (2) extracting all Si–O and Al–O bond lengths as well as Si–O–Si, Al–O–Al and Si–O–Al bond angles, followed by analyzing their statistical distributions and average values; (3) classifying Q^n^ species according to the number of bridging oxygens (*n* = 0–4) bonded to each Si atom and calculating their proportions to characterize the network connectivity; and (4) scanning the systems using probe spheres with a radius of 1.0 Å (approximately equal to the kinetic diameter of water molecules) via the grid method, identifying and counting the volume of connected cavities accessible to the probes; the ratio of this volume to the total system volume is defined as nanoporosity, which is used to evaluate the compactness of the network structure.

### 2.2. Experimental Scheme

#### 2.2.1. Raw Materials and Preparation of AAMs

Low-calcium fly ash (FA, CaO content < 10 wt%, Jinrun New Materials Co., Ltd., Zhengzhou, China) with a molar ratio of reactive SiO_2_ to Al_2_O_3_ of approximately 1:1 was employed as the primary aluminosilicate precursor in the experiments. Waste glass powder (GP, passed through a 600-mesh sieve, Zhuangwei New Materials Co., Ltd., Zhengzhou, China) and liquid sodium silicate (LSS, modulus 3.3, solid content ≥ 35.5%, Yourui Refractory Materials Co., Ltd., Jiashan County, China) were used as supplementary silica sources to precisely adjust the Si/Al ratio of the system. Sodium hydroxide pellets were adopted to provide additional alkalinity and regulate the modulus of sodium silicate. The detailed chemical compositions of fly ash and glass powder are presented in Table 3.

To ensure comparability with the simulations, four mixtures with molar silica-alumina (Si/Al) ratios of 1.0, 1.5, 1.8 and 2.0 were designed. The mix proportion design followed three principles: (1) maintaining a constant Na_2_O content (8 wt% of the total solid mass) to control the alkalinity, with other oxides (e.g., CaO, MgO, Fe_2_O_3_) excluded from this ratio calculation; (2) fixing the water-to-binder ratio at 0.4 to ensure workability and comparability; (3) adjusting the proportions of FA, GP and sodium silicate solution to achieve the target Si/Al ratios. The final Si/Al ratio of each system was calculated based on the total molar amounts of reactive SiO_2_ and Al_2_O_3_ contributed by all solid raw materials and sodium silicate solution, with the detailed mix proportions given in Table 4.

#### 2.2.2. Sample Preparation and Testing Methods

**Figure 2:**
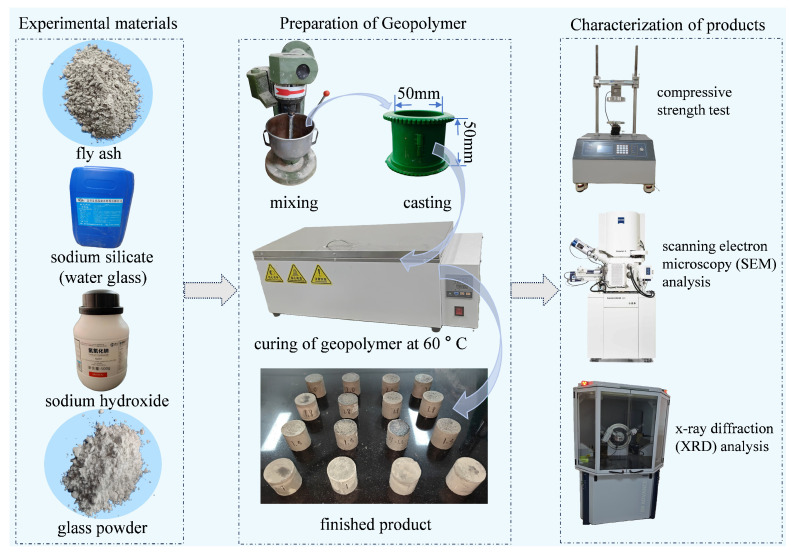
Experimental materials and methods.

Raw materials were weighed according to the mix proportions specified in Table 4. Sodium hydroxide (NaOH) was first dissolved in the calculated amount of water, and then mixed with sodium silicate solution to prepare the alkaline activator. Fly ash (FA) and glass powder (GP) were dry-mixed for 1 min, followed by the addition of the alkaline activator and mixing in a planetary mixer for 5 min to form a homogeneous paste. The paste was poured into molds with a diameter of 50 mm and a height of 50 mm, and vibrated to achieve full compaction. The samples, together with the molds, were sealed and cured in an oven at 60 °C for 24 h (elevating the temperature to 60 °C can significantly accelerate the reaction rate of fly ash without compromising the mechanical properties of the final reaction products [25]). After demolding, the samples were transferred to a standard curing room (20 ± 2 °C, RH > 95%) for further curing until the ages of 1 d, 3 d, 7 d and 28 d. Characterization tests were performed at the corresponding ages, with the detailed process flow shown in Figure 2.

The specific characterization methods for the reaction products are as follows:

X-ray Diffraction (XRD) Analysis: Ground powder samples were tested using an X-ray diffractometer (D8 ADVANCE, Bruker AXS GmbH, Fitchburg, WI, USA). The scanning range was 10–90° (2θ) with a step size of 0.02° and a scanning rate of 5°/min. Semi-quantitative phase analysis was conducted via the Rietveld full-spectrum fitting method.

Scanning Electron Microscopy (SEM): The morphology of samples was analyzed using a scanning electron microscope (EVO MA15, Carl Zeiss AG, Oberkochen, Germany) at an accelerating voltage of 15 kV. Prior to testing, the samples were gold-sputtered with a vacuum sputter coater, and a 5–10 nm thick gold film was deposited to enhance surface conductivity and prevent charge accumulation. The sample stage was kept stable during testing to ensure clear imaging and reliable results.

Unconfined Compressive Strength (UCS) Test: In accordance with ASTM C109 [32] and JTG 3441-2024 [33], the compressive strength of specimens was tested using an unconfined compressive strength testing machine (TKA-WXY-6F, Nanjing Tekao Technology Co., Ltd., Nanjing, China). The tests were performed at 25 °C with a loading rate of 5 mm/min. For each mix proportion, five parallel specimens were tested, and the results were averaged.

## 3. Results and Discussion

### 3.1. Microstructural Evolution Law Revealed by Molecular Dynamics Simulation

#### 3.1.1. Gelation Kinetics and the Dominant Role of Aluminum Coordination

**Figure 3:**
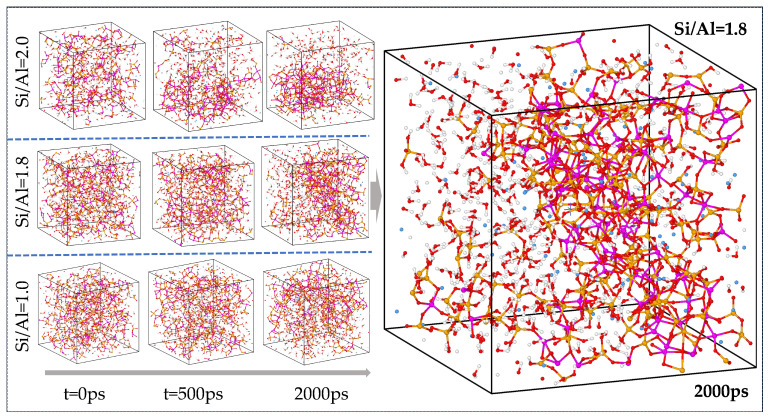
Structural evolution of N A S H gel for Si/Al ratios.

The simulation successfully reproduced the dynamic process from discrete PS/PSS oligomers to a continuous gel network, as illustrated in Figure 3. In the construction of the N-A-S-H gel model, all initial aluminosilicate species existed as PS and PSS oligomers. The essence of their polymerization reaction lies in the process where PS and PSS oligomers form bridging oxygen (Q^n^) crosslinking sites via dehydration of OH and H groups, accompanied by water molecule release. As the reaction proceeded, these structural units underwent continuous condensation to form the aluminosilicate network skeleton. When the reaction proceeded to 2000 ps, the aluminosilicate chains exhibited low dispersion in the system with Si/Al = 1.0, whereas the chain dispersion increased significantly under the condition of Si/Al = 2.0.

**Figure 4:**
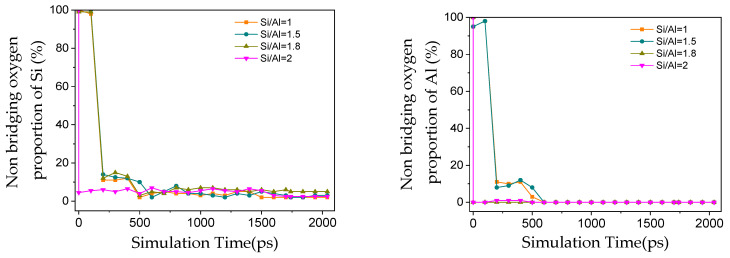
Evolution of non-bridging oxygen proportion for silicon and aluminum species with reaction time under different Si/Al ratios.

**Figure 5:**
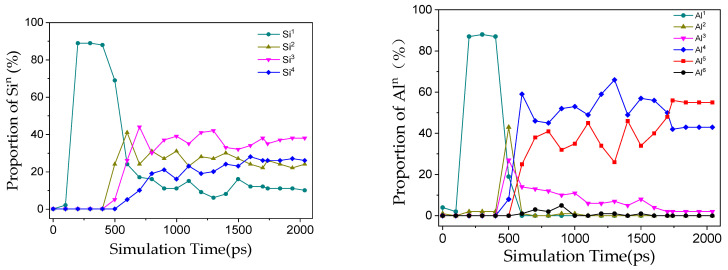
Evolution of Q^n^ species during the condensation reaction (Si/Al = 1.0).

By tracking the decay in the number of non-bridging oxygens (Si–OH and Al–OH) and the content variation in newly formed Q^n^ species (Figure 4 and Figure 5), it was found that the consumption rate of Al–OH was faster than that of Si–OH under all Si/Al conditions, which kinetically confirms the higher reactivity of Al species. This is consistent with the theoretical understanding that the dissociation energy of Al–O bonds is generally lower than that of Si–O bonds, and also explains the common phenomenon of preferential Al dissolution observed in experiments. As the simulation proceeded, non-bridging oxygens were continuously converted into Q^n^ species with higher polymerization degrees (Si^n^ and Al^n^, n = 1–4): Si^1^ appeared first, followed by the sequential formation of Si^2^, Si^3^ and Si^4^, indicating that the reaction follows a stepwise polymerization pathway from monomers to oligomers and then to large clusters, which is consistent with the actual geopolymerization process [34]. At the end of the simulation, most non-bridging oxygens were consumed, with only a small amount remaining due to intrinsic topological defects in the amorphous network (e.g., broken bonds and under-coordinated sites). These defects render the network more susceptible to OH^−^ attack and promote the preferential formation of Si^3^ during depolymerization, resulting in the highest proportion of Si^3^ in the end. The evolution of the aluminooxygen network was similar to that of the silicooxygen network; Al–OH was almost completely consumed eventually, but a considerable proportion of pentacoordinate aluminum (Al^5^) and a small amount of hexacoordinate aluminum (Al^6^) were formed during the process [35]—the defect sites in the amorphous network provide a stable environment for Al^5^, leading to its final proportion exceeding 50%.

**Figure 6:**
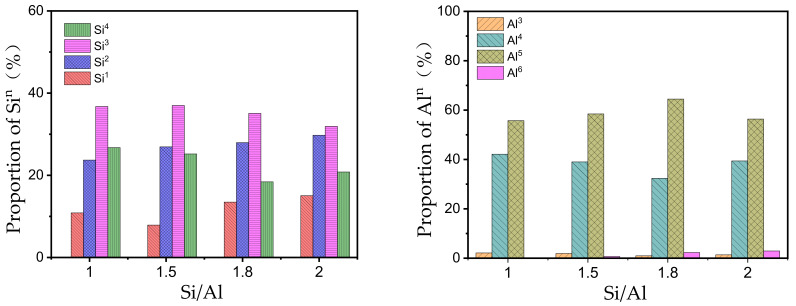
Distribution of Si^n^ and Al^n^ sites in N-A-S-H gel models at different Si/Al ratios.

Coordination analysis of the final gel structure reveals a striking feature that deviates from conventional understanding: pentacoordinate aluminum (Al^5^) dominates as the primary Al species across all four Si/Al systems, with its proportion stably exceeding 50% of the total aluminum content (Figure 6). In contrast, the classic tetracoordinate aluminum (Al^4^) accounts for approximately 30–40%, along with a small amount of hexacoordinate aluminum (Al^6^). This result differs from numerous early simulations based on ideal tetrahedral models and some NMR studies, but is consistent with recent advanced characterization findings under high-alkalinity and non-equilibrium synthesis conditions (e.g., the works by Wang Z et al. [36,37,38]) as well as the trends of several latest reactive force field simulations [11,39]. We propose that in the dynamic, water-rich strong alkaline environment depicted by ReaxFF simulations, Al^5^ is not a “defect” but rather a key intermediate stable state or charge regulator. Its formation stems from two aspects: on the one hand, the strong affinity of Al for hydroxyl ligands under high pH values; on the other hand, it may serve to balance local structural stress and negative charges more effectively, especially when Na^+^ exhibits uneven spatial distribution. Al^5^ sites bear more hydroxyl groups and may act as an “active reservoir” for condensation reactions, facilitating the rapid growth of the gel network.

#### 3.1.2. Network Topology and Bonding Environment

The connection state of silicon-oxygen tetrahedra (Q^n^ distribution) is a key indicator for evaluating network polymerization degree. Specifically, Q^1^ represents terminal sites, Q^2^ denotes middle groups of chain/cyclic structures, Q^3^ refers to branched structures, and Q^4^ indicates fully cross-linked structures [40,41]; the overall structural composition can be inferred from their distribution proportions. As shown in Figure 6, a considerable proportion of high-connectivity species (Q^3^ + Q^4^, total 53–64%) were generated in all systems, confirming the formation of three-dimensional (3D) networks. Notably, as the Si/Al ratio increased from 1.0 to 2.0, the ratio of (Q^3^ + Q^4^)/(Q^1^ + Q^2^) exhibited a decreasing trend. This indicates that lower Si/Al ratios (1.0, 1.5) are more conducive to the formation of highly cross-linked networks, whereas higher Si/Al ratios (1.8, 2.0) may lead the network connectivity topology to tend toward lower dimensionality or more chain-like structures. This is consistent with the chemical intuition that an increase in Si/Al ratio results in a relative reduction in Al sites available for cross-linking.

**Figure 7:**
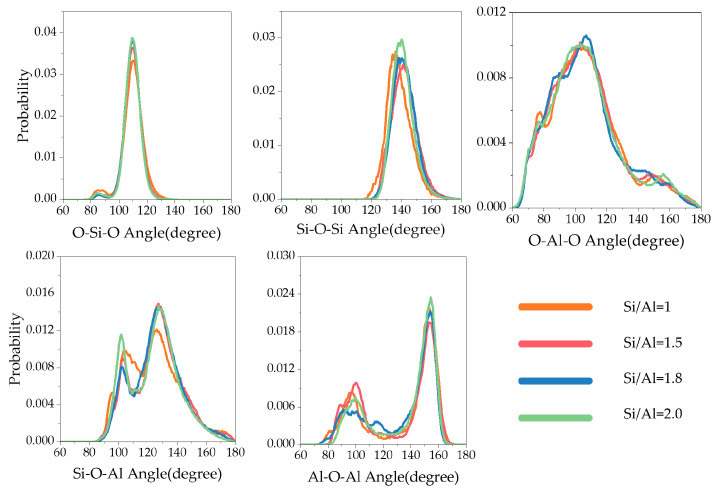
Bond angle distribution in the N-A-S-H Gel model.

Bond angle distribution provides more refined structural information (Figure 7). The Si-O-Si bond angle exhibited a unimodal distribution with a peak at approximately 140° and a narrow distribution range, reflecting the intrinsic rigidity of [SiO_4_]^4−^ tetrahedral connections. In contrast, the Al-O-Al bond angle showed a unique bimodal distribution (~100° and ~150°), which directly reflects the significant local structural distortion caused by aluminum coordination polymorphism (coexistence of Al^4^ and Al^5^/Al^6^). This flexibility or “disorder degree” of bond angles is an important feature that distinguishes N-A-S-H gels from crystalline zeolites, and may also serve as potential weak points for stress concentration.

**Figure 8:**
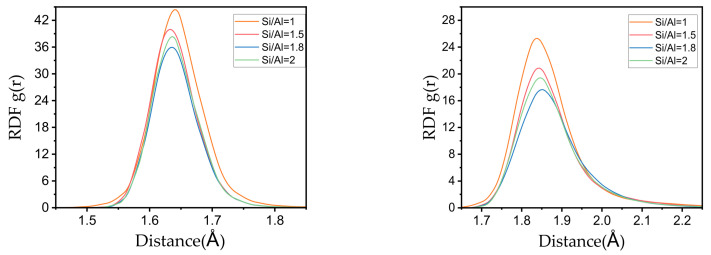
Bond length distribution of Si-O (**left**) and Al-O (**right**) in the N-A-S-H Gel model.

Bond length distribution reveals the fundamental linkage characteristics of the gel network (Figure 8). The simulated average Si–O bond length (1.64 Å) is in good agreement with reported literature values, while the average Al–O bond length (1.84 Å) is systematically higher [12,42]. This difference mainly stems from the high proportion of pentacoordinate aluminum (Al^5^) predicted by this model; the Al–O bond length of Al^5^ is inherently longer than that of common tetracoordinate aluminum (Al^4^), thus elevating the overall average value. In addition, as the Si/Al ratio increases from 1.0 to 2.0, both Si–O and Al–O bond length distributions show a slight right shift, indicating a corresponding increase in average bond length. This trend is consistent with existing studies and explains the macroscale mechanism by which increasing Si/Al ratio reduces the elastic modulus of N-A-S-H gels from an atomic perspective—longer bond lengths generally correspond to weaker bonding strength and lower network stiffness.

#### 3.1.3. Non-Monotonic Nanoporosity Variation and Optimal Si/Al Ratio

**Figure 9:**
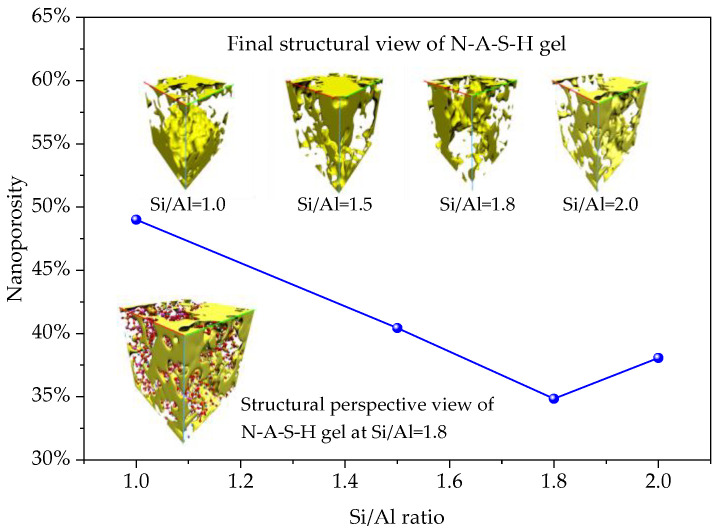
Formation and distribution of nanopores in N-A-S-H gel models at different Si/Al ratios, where yellow indicates pore regions.

The calculated nanoporosity (probe radius: 1.0 Å) exhibited a clear non-monotonic trend of first decreasing and then increasing with Si/Al ratio (Figure 1e). It decreased gradually from 49.3% at Si/Al = 1.0, reached a minimum of 35.2% at Si/Al = 1.8, and then rose back to 42.1% at Si/Al = 2.0, as shown in Figure 9. The existence of this inflection point is crucial, indicating that the atomic packing density of the gel network does not improve monotonically with increasing Si content but has an optimal stoichiometric balance point (~1.8).

The mechanism underlying this phenomenon is explained as follows: In the low Si/Al region (≤1.8), increasing Si content provides more [SiO_4_]^4−^ tetrahedral units for skeleton construction, which combine with sufficient Al cross-linking sites to form a more complete and compact network, leading to decreased porosity. At Si/Al = 1.8, the Si/Al ratio achieves optimal synergy, enabling sufficient network cross-linking, robust skeleton, high Na^+^ charge compensation efficiency, the densest structure and optimal mechanical properties. When the Si/Al ratio further increases (>1.8), the relative shortage of Al atoms available for effective cross-linking causes excessive Si to tend to form longer linear Si–O–Si segments with low packing efficiency. Meanwhile, the high proportion of Al^5^/Al^6^ requires more isolated Na^+^ compensation, and these hydrated Na^+^ ions occupy extra space. Together, these factors result in increased nanoscale free volume and thus porosity rebound.

### 3.2. Experimental Validation: Correlation Between Macroscopic Properties and Microstructure

#### 3.2.1. Mechanical Property Response to Si/Al Ratio

**Figure 10:**
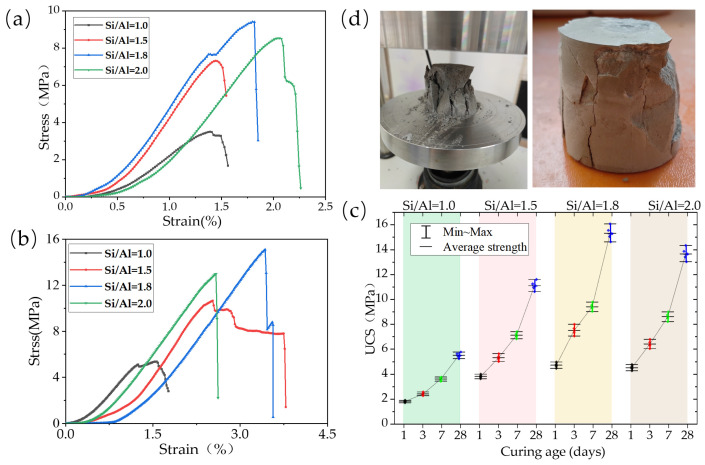
Evolution of mechanical properties: (**a**,**b**) stress–strain curves at 7 and 28 days of curing, respectively; (**c**) development of compressive strength over different curing ages (1, 3, 7, and 28 days). (**d**) Representing the failure morphology of the specimen.

The experimentally measured unconfined compressive strength (UCS) results (Figure 10) provide strong macroscopic evidence for the simulation predictions. The specimens exhibited predominantly brittle fracture, and the strength variation curve with Si/Al ratio exhibits a distinct peak characteristic. Statistical analysis showed that both curing age and Si/Al ratio had an extremely significant effect on UCS (*p* < 0.0001), and the strength values of the Si/Al = 1.8 group were significantly higher than those of other groups. Specimens with Si/Al = 1.8 achieved the highest strength at both 7 d and 28 d (specifically: average value 9.4 MPa, standard deviation 0.283 MPa, 95% confidence interval [9.05, 9.75] MPa; and average value 15.3 MPa, standard deviation 0.539 MPa, 95% confidence interval [14.64, 15.98] MPa). Statistical analysis (one-way ANOVA, *p* < 0.05) indicated that the strength values of this group were significantly higher than those of other groups. For specimens with Si/Al = 1.0, insufficient silicon source resulted in a limited total amount of cementitious phases formed by the reaction, leading to a loose structure and the lowest strength. Meanwhile, the strength of specimens with Si/Al = 2.0 decreased compared to the Si/Al = 1.8 group, which is fully consistent with the simulation predictions of increased porosity and reduced structural compactness at this ratio. The inverse correlation between macroscopic strength and simulated nanoporosity establishes a key bridge from atomic-scale structure to macroscopic properties.

#### 3.2.2. Evidence of Phase Evolution and Microscopic Morphology

**Figure 11:**
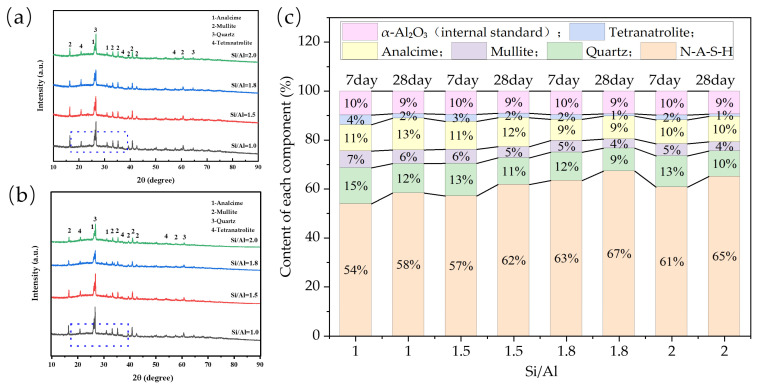
XRD patterns and phase content of samples with different Si/Al ratios at 7 and 28 days. (**a**) 7 d XRD patterns of specimens with different Si/Al ratios; (**b**) 28 d XRD patterns of specimens with different Si/Al ratios; (**c**) Quantitative analysis results.

X-ray diffraction (XRD) combined with Rietveld refinement was employed for quantitative phase analysis of alkali-activated cementitious materials at 7 d and 28 d curing ages. The results (Figure 11c) showed that as the Si/Al ratio increased from 1.0 to 1.8, the contents of quartz and mullite in the raw materials decreased continuously, while the content of amorphous N-A-S-H gel product increased correspondingly (The parts enclosed by the blue dashed boxes in Figure 11a,b), indicating a gradual improvement in reaction degree. At Si/Al = 1.8, the residual raw materials were minimized and the gel product was maximized, corresponding to the highest reactivity and conversion rate. When the Si/Al ratio further increased to 2.0, the reaction degree decreased slightly, which may be related to the excess silicon altering the liquid-phase chemical equilibrium and affecting precursor dissolution and condensation kinetics.

XRD data further revealed the dynamic phase evolution during the reaction: in the alkaline environment, crystalline phases such as quartz and mullite continuously dissolved, releasing reactive aluminosilicate species that formed amorphous N-A-S-H gel via condensation. This gel gradually encapsulated unreacted particles. As the reaction proceeded, metastable Tetranatrolite precipitated from part of the gel or liquid phase; in the long-term reaction, it gradually dissolved and transformed into thermodynamically more stable analcime crystals via the Ostwald ripening mechanism, leading to a decrease in the content of the former and a significant increase in the latter. Meanwhile, the three-dimensional (3D) amorphous gel network formed by the condensation of aluminosilicate oligomers continued to develop and densify. Eventually, the system evolved into a composite structure consisting of amorphous N-A-S-H gel and stable zeolite crystals (predominantly analcime). The formation of crystalline phases helps improve the long-term chemical stability and compactness of the system, and is one of the key factors for geopolymers to achieve long-term strength, thus explaining the highest strength of specimens with Si/Al = 1.8 at the mesoscopic scale.

**Figure 12:**
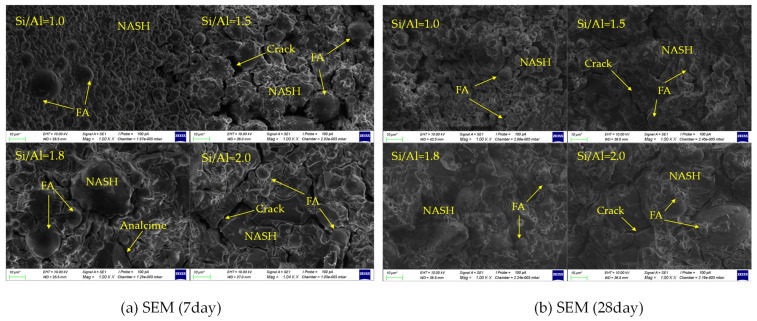
SEM Images of Geopolymer Products with Different Si/Al Molar Ratios.

Scanning electron microscopy (SEM) observations (Figure 12) provided more intuitive microstructural evidence. In samples with Si/Al = 1.0, a large number of unreacted fly ash spherical particles were dispersed in the loose gel matrix. For samples with Si/Al = 1.5 and 1.8, the number of unreacted particles decreased, and the gel phase became more continuous and dense. Especially in the sample with Si/Al = 1.8, the gel exhibited a uniform and dense morphology with few pores. In contrast, although the number of unreacted particles was also small in the sample with Si/Al = 2.0, some microcracks and finer pores appeared in the gel phase, resulting in slightly poor structural uniformity. These observations directly support the conclusion from simulations that the optimal dense structure occurs around Si/Al = 1.8.

### 3.3. Comprehensive Discussion: Regulation Mechanism of Si/Al Ratio and Structure-Property Relationship

Based on the in-depth mutual verification of the aforementioned simulation and experimental results, we propose an integrated mechanism for the regulation of N-A-S-H gel structure and properties by Si/Al ratio:

1. Al-rich region (Si/Al < 1.8): Sufficient Al species can rapidly form cross-linking sites, promoting the three-dimensionalization of the network. However, the silicon skeleton is relatively weak, and a large amount of Al may exist in high-coordination states (Al^5^, Al^6^), requiring more Na^+^ for charge compensation. Insufficient compensation or large steric hindrance tends to form local loose structural regions. The final structure has a moderate degree of cross-linking but inherently high porosity, corresponding to moderate macroscopic strength.
2. Optimal region (Si/Al ≈ 1.8): The Si/Al ratio achieves an optimal match between thermodynamics and kinetics. Al provides moderate cross-linking sites, while Si forms a robust and moderately branched skeleton. Al^5^ exists as an effective charge buffer and reaction intermediate, achieving efficient synergy with Na^+^. Under this condition, the condensation reaction degree is high, forming an ideal gel network with high cross-linking, low porosity, and high continuity, corresponding to the peak macroscopic strength.
3. Si-rich region (Si/Al > 1.8): Al cross-linking sites are relatively scarce, and Si tends to polymerize into long-chain structures, leading to a decrease in network cross-linking density. Limited Al mostly exists in high-coordination states to balance the system charge, exacerbating structural disorder. Long-chain silicates have poor close packing ability, and the isolation effect of Na^+^ is more significant. Together, these factors result in a significant rebound in nanoporosity and a decrease in the overall structural stiffness and strength of the gel.

The core of this mechanism lies in the synergy and competition between “aluminum coordination polymorphism” and “silicate polymerization degree.” By regulating the balance of these two factors, the Si/Al ratio ultimately determines the nanoscale topology and pore characteristics of the gel network, thereby controlling the macroscopic mechanical properties. In this study, the key role of Al^5^, the non-monotonic variation in nanoporosity, and its optimal value at Si/Al = 1.8 collectively constitute the microscopic evidence chain for this regulation mechanism.

Although this study reveals the regulation mechanism of the Si/Al ratio on N-A-S-H gels, its in-depth understanding is still limited by the size and time scale of the computational model, force field accuracy, simplified chemical composition (Ca, Fe and other elements not considered), and the coupling effect of experimental variables. Future research can develop cross-scale computational methods combined with multi-variable collaborative experiments to achieve more accurate prediction and design of material properties in systems closer to real conditions.

## 4. Conclusions

In this study, by integrating reactive molecular dynamics simulations and systematic experimental studies, and spanning atomic–nano–micro–macro scales, we profoundly revealed the regulation laws and mechanisms of the Si/Al ratio on the structure and properties of alkali-activated N-A-S-H gels, and obtained the following main conclusions:

1. Revealed the dominant role of pentacoordinate aluminum: In the dynamic condensation environment, pentacoordinate aluminum (Al^5^) is the main existing form of aluminum in N-A-S-H gels (>50%). It may play a key role in charge balance and reaction promotion, which updates the understanding of the local structure of gels.
2. Discovered the optimal ratio for structural densification: Molecular simulations clearly show that the nanoporosity of the gel reaches a minimum (35.2%) at Si/Al ≈ 1.8, indicating the highest atomic packing efficiency and optimal network compactness at this ratio.
3. Established a cross-scale structure-property relationship: Experiments confirmed that the specimen with Si/Al = 1.8 exhibits the highest reaction degree, the most uniform and dense microscopic morphology, and the highest macroscopic compressive strength (15.3 MPa at 28 d), which perfectly verifies the simulation predictions and establishes a strong correlation between nanoporosity and macroscopic strength.
4. Clarified the regulation mechanism of Si/Al ratio: This study proposes that the Si/Al ratio regulates the gel structure by regulating the balance between “Al cross-linking capacity” and “Si skeleton construction capacity”. Deviation from the optimal ratio will lead to insufficient cross-linking or excessive skeleton polymerization, thereby causing an increase in nanoporosity and performance degradation.

This study not only provides atomic-scale insights into understanding the intrinsic origin of performance of alkali-activated materials, but more importantly, the revealed optimal Si/Al ratio (~1.8) and related structural characteristic parameters (low porosity, appropriate Al^5^ proportion) can offer theoretical guidance and quantitative basis for the rational design and precise regulation of alkali-activated cementitious materials targeting high-performance requirements.

## Figures and Tables

**Table 1:** Initial Compositions and Parameters of the Molecular Dynamics Simulation System.

System ID	Target Si/Al Ratio	Number of PS	Number of PSS	Number of Na^+^ (NaOH)	Initial H_2_O Molecules	Final H_2_O Molecules	Total Density (g/cm^3^)
GP-1.0	1	100	0	100	473	694	1.443
GP-1.5	1.5	50	50	100	560	807	1.708
GP-1.8	1.8	20	80	100	612	848	1.867
GP-2.0	2	0	100	100	647	898	1.973

**Table 2:** Simulation Process.

Step Number	Simulation Process	Ensemble Type	Temperature Condition	Time Parameter
①	Energy Minimization	—	—	—
②	Initial System Equilibration	NVT	300 K	100 ps
③	Density Equilibration	NPT	300 K	300 ps
④	Heating Process	NVT	300 K rise to 2000 K (17 K/ps)	100 ps
⑤	High-Temperature Relaxation	NVT	2000 K	1000 ps
⑥	Quenching Process	NVT	2000 K drop to 300 K (5 K/ps)	340 ps
⑦	Room-Temperature Relaxation	NVT	300 K	300 ps

**Table 3:** Material Composition Table.

Chemical Components (%)	SiO_2_	Al_2_O_3_	CaO	Na_2_O	MgO	Fe_2_O_3_	Others
Fly Ash	45.10	36.80	4.50	0	0	0.85	12.75
Glass Powder	71.58	0.81	4.41	13.8	3.94	0	5.46

**Table 4:** Mix Proportion Design of Experimental Samples (Parts by Mass).

Sample ID	Target Si/Al	FA	GP	LSS	NaOH	Add. Water	Calculated Si/Al
E-1.0	1	500	0	0	51.6	150	1.02
E-1.5	1.5	500	0	367	11.2	95	1.51
E-1.8	1.8	500	52	469	0	70	1.79
E-2.0	2	500	112	469	0	60	2.03

## Data Availability

The original contributions presented in this study are included in the article. Further inquiries can be directed to the corresponding authors.
